# Effectiveness of exercise training on arterial stiffness and blood pressure among postmenopausal women: a systematic review and meta-analysis

**DOI:** 10.1186/s13643-024-02589-y

**Published:** 2024-07-02

**Authors:** Deshuang Yang, Shiyi Tao, Mingjing Shao, Li Huang, Xiang Xiao, Jin Zhang, Ruiqi Yao, Ziyi Sun

**Affiliations:** 1 Department of Integrative Cardiology, China-Japan Friendship Hospital, Beijing, China; 2 Graduate School, Beijing University of Chinese Medicine, Beijing, China; 3 Department of Internal Medicine, Shenzhen Nanshan Chinese Medicine Hospital, Guangdong, China

**Keywords:** Exercise training, Arterial stiffness, Postmenopausal women, Hypertension

## Abstract

**Background:**

The acute and long-term benefits of exercise training on cardiovascular health have been well established. The systematic review and meta-analysis aimed to systematically assess the effectiveness of exercise training on arterial stiffness and blood pressure among postmenopausal women with elevated blood pressure.

**Methods:**

A comprehensive search was conducted on PubMed, Embase, Web of Science, ProQuest, Cochrane Library, Cochrane Central Register of Controlled Trials, and ClinicalTrials.gov website from inception to September 30, 2023, to identify the randomized controlled trials (RCTs), which evaluated the effectiveness of exercise training on arterial stiffness and blood pressure in postmenopausal women. Standardized mean differences (SMD), weighted mean differences (WMD), and 95% confidence intervals (95% CIs) were calculated using random/fixed effects models. Quality assessment was performed using the modified Jadad scale and the Cochrane Risk of Bias Tool. Sensitivity analysis and subgroup analysis were conducted based on drug dosage, treatment duration, and age of administration to further explore potential heterogeneity. Funnel plots were performed to assess publication bias and Begg’s regression test was carried out for funnel plot asymmetry.

**Results:**

Twenty-two RCTs involving 1978 participants were included in the quantitative analysis. The mean quality of eligible studies was 4.2 out of 7 based on the modified Jadad scale. The results indicated that exercise training had a significant effect on reducing brachial-ankle pulse wave velocity [MD = − 0.69, 95%CI (− 1.11, − 0.27), *P* = 0.001], decreasing augmentation index (AIx) [MD = − 6.00, 95%CI (− 6.39, − 5.61), *P* < 0.00001] and AIx normalized to a heart rate of 75 beats per minute (AIx@75%) [MD = − 7.01, 95%CI − 7.91 to − 6.12, *P* < 0.00001], lowering systolic blood pressure [MD = − 6.19, 95%CI − 9.24 to − 3.15, *P* < 0.0001], diastolic blood pressure [MD = − 3.57, 95%CI (− 6.10, − 1.03), *P* = 0.006) and pulse pressure [MD = − 8.52, 95%CI (− 16.27, − 0.76), *P* = 0.03]. Subgroup analysis revealed that baseline blood pressure levels had a large impact on the effect of exercise training.

**Conclusions:**

The systematic review and meta-analysis suggested that exercise training may ameliorate arterial stiffness and reduce blood pressure in postmenopausal women with elevated blood pressure. However, the optimal mode of exercise training that improves arterial stiffness and blood pressure in this population requires further investigation.

**Systematic review registration:**

PROSPERO CRD42021211268

**Supplementary Information:**

The online version contains supplementary material available at 10.1186/s13643-024-02589-y.

## Introduction

The incidence of hypertension is significantly higher in postmenopausal women [1, 2]. Menopause is one of the risk factors for cardiovascular diseases [3], with sex hormone deficiency [4], endothelial dysfunction [5], and arterial stiffness [6]. It has been proved that estrogen plays an important role in inducing the mobilization of endothelial progenitor cells in the bone marrow to promote angiogenesis and repair endothelial damage [7]. However, estrogen deficiency reduces the repair capacity of endothelial cells, ultimately leading to arterial damage and endothelial dysfunction in older women [4, 8, 9]. Abnormal blood pressure may result from decreased arterial compliance associated with endothelial dysfunction [10, 11]. Arterial stiffness is widely regarded as an important predictor and potential therapeutic target for hypertensive patients, with prognostic value for cardiovascular disease [12–15]. Measurements of arterial stiffness have been recommended as a valuable method in the preventive management of cardiovascular disease [16]. Thereinto, the brachial-ankle pulse wave velocity (baPWV) and augmentation index (AIx) are two important indicators for evaluating arterial stiffness [17, 18].

Adverse reactions such as hypokalaemia, glucose intolerance, and dry cough may occur during the use of antihypertensive drugs, which may reduce patient compliance and decrease treatment effectiveness [16]. The risk of cardiovascular disease in hypertensive patients cannot be reduced to the same level as in healthy people even with rigorous blood pressure treatment [19, 20]. The study found an unsatisfactory effect of antihypertensive drugs on arterial stiffness and PWV [21]. Hormone replacement therapy is common in postmenopausal women, but the improvement in arterial stiffness with standard hormone replacement therapy has not been observed [22]. Lifestyle modifications including exercise training have been recommended by the International Society of Hypertension (ISH) guidelines as the preferred intervention before medications in hypertensive patients [23]. Substantial studies have shown that exercise can not only lower blood pressure but also reduce blood lipid levels [24] and enhance cardiac function [25]. Aerobic and resistance exercise training might be beneficial for the prevention and treatment of hypertension and arterial stiffness [26–28]. Data from a randomized control trial (RCT) revealed that stair climbing led to reductions in arterial stiffness, blood pressure, and increases in leg strength in stage 2 hypertensive postmenopausal women [29]. Moreover, exercise training for 12 weeks (180 min per week) improved arterial stiffness in elder women with hypertension [30]. Previous studies have found that aerobic and resistance exercise training improved arterial stiffness and lowered blood pressure in postmenopausal women with elevated blood pressure [29–31]. Nevertheless, similar effects were not observed in several other studies [32–35]. Thus, the potential effects of exercise training on arterial stiffness and blood pressure in postmenopausal women with elevated blood pressure need to be well understood.

The small sample size of these studies may account for the observed differences. Meta-analysis plays a role in comprehensive evaluation by summarizing the results of multiple studies with small sample sizes and performing systematic analysis. The essence of this synthesis is equivalent to increasing the sample size to achieve the purpose of improving the estimation of the effect size. To our knowledge, no previous meta-analyses have been performed to examine the comprehensive effect of different exercise training on arterial stiffness and blood pressure in this population. Therefore, this study conducted a systematic review and meta-analysis to systematically assess the exercise effects on arterial stiffness and blood pressure in postmenopausal women with elevated blood pressure.

## Methods

### Protocol and registration

The systematic review and meta-analysis followed the Preferred Reporting Items for Systematic Reviews and Meta-Analysis (PRISMA) guidelines (Additional file 1) and the protocol has been recorded in PROSPERO (CRD42021211268).

### Search strategy

A systematic search was conducted according to the PRISMA statement up to September 30, 2023, using PubMed, Embase, Web of Science, ProQuest, Cochrane Library, ClinicalTrials.gov website (https://clinicaltrials.gov/), and Cochrane Central Register of Controlled Trials (https://www.cochranelibrary.com) to identify eligible randomized trials. In addition, we obtained the references of published studies by manually retrieving, personal communication and other sources. The search terms included “postmenopausal”, “arterial stiffness”, “blood pressure”, “vascular stiffness”, “pulse wave velocity”, “augmentation index”, “pulse pressure” (Additional file 2).

### Inclusion and exclusion criteria

We included the following studies: (1) postmenopausal women enrolled in the RCTs were diagnosed with elevated blood pressure based on the 2017 ACA/AHA Guidelines [36]. Postmenopausal women are defined as women with amenorrhea for at least 1 year and/or serum follicle stimulating hormone (FSH) concentration > 40 miU/ml, or women with a history of hysterectomy and bilateral oophorectomy [37]. (2) The intervention group underwent aerobic exercise training, resistance exercise training, or combined exercise training, and the participants were asked to complete the whole exercise course. Participants in the control group were instructed to maintain regular lifestyle habits, keep sedentary, and receive sham training or entertainment programs. Discontinued hormone replacement therapy or have been on stable hormone replacement therapy with or without hypotensive drugs for at least 1 year, and remain unchanged during exercise training. (3) Studies with at least one of the following outcomes: baPWV, systolic blood pressure (SBP), diastolic blood pressure (DBP), PP, AIx, and AIx normalized to a heart rate of 75 beats per minute (AIx@75%).

Excluded studies were as follows: (1) severe comorbidities that make exercise training intolerant during treatment. (2) Data were incomplete or inconsistent, or the full text of the literature could not be obtained. (3) Only one study with the most complete data was included for duplications.

### Study selection

Data selection was independently performed by two researchers (DSY and SYT) using EndNote X9 reference management software. After eliminating repetitive literature, the titles and abstracts of all potentially relevant studies were independently examined and the full-text records were retrieved for eligibility, followed by a full-text review. Disagreement on inclusion was resolved by consensus and after discussion with the senior reviewer.

### Data extraction

The data was independently extracted by DSY and SYT into an Excel table, including study information (first author, publication year, country, years of collection, registration number, sample size), patient demographics (age, gender), interventions, and the outcomes (baPWV, AIx, AIx@75%, SBP, DBP, PP, and adverse events). Finally, two researchers cross-checked the entered information, and disagreement on information was resolved by consensus after checking with the original studies.

### Quality assessment

Quality assessment was conducted independently by two reviewers (DSY and SYT) using the modified Jadad scale. Any disagreement in opinion regarding quality was resolved by discussion consensus with a third investigator (MJS). The modified Jadad scale contains 5 items for RCTs, with a score ranging from 0 to 7: randomization, allocation concealment, blinding, and dropout/withdrawal. A score of 1 to 3 indicates low quality, whereas a score of 4 or more indicates high quality [38]. Cochrane Handbook 5.1.0 [39] was used for assessing the quality of RCTs whereby evaluated the random sequence generation, allocation concealment, blinding of participants and personnel, blinding of outcome assessment, incomplete outcome data, selective outcome reporting, and other sources of bias. Each trial was ranked as having an unclear, high, or low risk of bias for each item.

### Statistical analysis

The meta-analysis was performed using Review Manager (version 5.3.0, Cochrane Collaboration, The Nordic Cochrane Center, Copenhagen) and Stata 12.0. Meta-analysis was conducted if two or more studies provided the same effect concerning the outcomes. Adjusted mean difference (MD) along with their respective standard deviation (SD) were extracted from each of the studies, and each effect size was expressed in a 95% confidence interval (95% CI). Inter-study heterogeneity was evaluated using Cochran’s *Q* statistics and *I*^2^-test. Low heterogeneity was defined as an *I*^2^ value less than 25%, moderate heterogeneity as a value of 25 ~ 50%, and high heterogeneity as a value larger than 50%. Heterogeneity was considered significant as either *P* < 0.10 and *I*^2^ > 50%, prompting a random-effects modeling estimate. Otherwise, a fixed-effects approach was used. Sensitivity analysis was considered to examine the influence of each study on the stability of the meta-analysis results. Subgroup analyses were attempted to address potential sources of heterogeneity. Funnel plots were conducted to assess the publication bias of indicators with more than 10 included studies. Furthermore, we also performed Begg’s regression test for funnel plot asymmetry, to verify whether the association between effect sizes and the related standard error was statistically significant.

## Results

### Search results

**Fig. 1:**
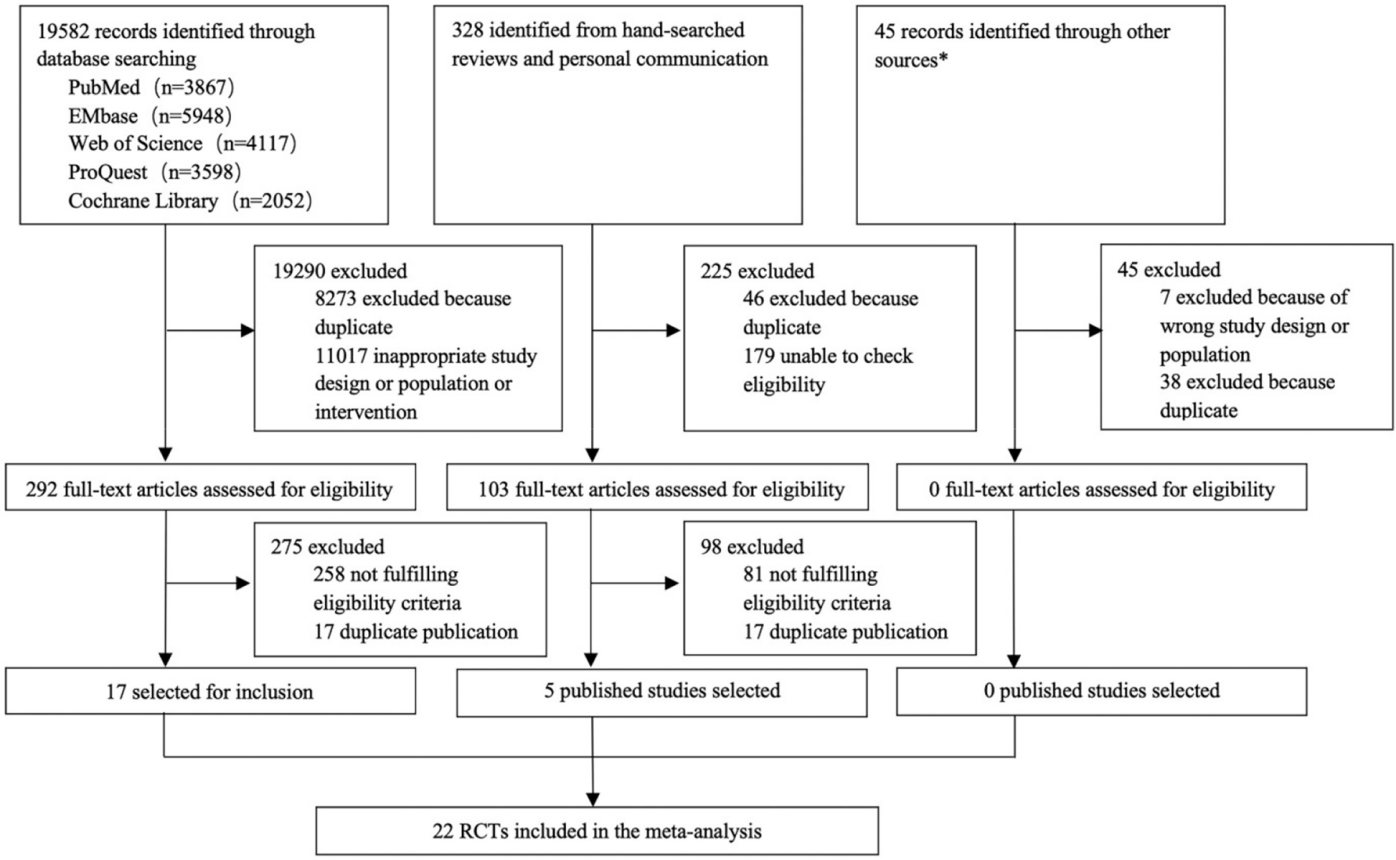
Study selection process. RCTs, randomized controlled trials. *Industry websites, contact with authors, and trial registries

Nineteen thousand nine hundred fifty-five references were identified, including 19,582 trials from the database search, 328 studies from personal communication or hand-searching other review articles, and 45 from trial registries or other sources. Three hundred ninety-five potentially eligible articles were retrieved in full text, of which 22 parallel RCTs were included in the meta-analysis. Figure 1 illustrates the different phases of the search and selection processes.

### Study characteristics

As shown in Table 1, among the 22 eligible studies, the efficacy and safety of three exercise types involving aerobic exercise (*n* = 18), resistance exercise (*n* = 2), and combined exercise (*n* = 2) on arterial stiffness and blood pressure in postmenopausal women with elevated blood pressure were examined. Duration of exercise training varied from 6 to 24 weeks. Trials were published from 2001 to 2020 and carried out in the USA, Korea, Canada, Egypt, Turkey, Japan, and Iran. A total of 1978 patients were retrieved in this study, of which 1390 participants were randomly assigned to an exercise training group and 588 were divided into the control group. The number of patients included in these studies ranged from 8 to 464. Taking into account different diseases and clinical conditions, was comprised of postmenopausal women with sedentary, untreated prehypertension, stage 1 hypertension, and stage 2 hypertension.

**Table 1:** Characteristics of eligible studies

Author	Year	Country	Registration number	Simple size		Age		Intervention		Training type	Training frequency	Duration/w	Outcomes	Jadad scale
				T	C	T	C	T	C					
A Wong et al	2014	USA	Clinical Trials.gov (NCT01741766)	14	14	57 ± 3.74	56 ± 3.74	Stretching training	RH	aerobic exercise training	3 times per week	8	①②③④⑤	4
Alexei Wong et al	2016	USA	Clinical Trials.gov (NCT02143817)	13	12	58 ± 3.61	58 ± 3.46	WBV training	RH	aerobic exercise training	3 times per week	8	④⑤	5
Alexei Wong-a et al	2018	USA	Clinical Trials.gov (NCT03254251)	20	21	59 ± 4.58	59 ± 4.47	Stair climbing	RH	aerobic exercise training	4 times per week	12	①④⑤	4
Alexei Wong-b et al	2018	USA	Clinical Trials.gov (NCT03546270)	52	48	75 ± 3	74 ± 4	SWM training	NE	aerobic exercise training	3 or 4 times per week	20	②③④⑤	7
Arturo Figueroa-a et al	2014	USA	Clinical Trials.gov (NCT01741779)	15	13	56 ± 3	56 ± 3	WBV training	NE	aerobic exercise training	3 times per week	6	②③④⑤⑥	3
Arturo Figueroa-b et al	2014	USA	Clinical Trials.gov (NCT01741779)	24	12	57 ± 3.53	58 ± 3.46	WBV training	NE	aerobic exercise training	3 times per week	12	①④	5
Benoit J Arsenault et al	2009	Canada	NR	267	82	57.3 ± 6.6	57.2 ± 6.1	Cycle and treadmill	NE	aerobic exercise training	3 or 4 times per week	24	④⑤	3
Beth A Staffileno et al	2001	CUSA	NR	9	9	57.1 ± 8.7	62.3 ± 8.7	Pleasurable activities	RH	aerobic exercise training	15 times per week	8	④⑤	3
Damon L Swift-a et al	2012	USA	NR	132	23	57.4 ± 5.8	56.8 ± 5.4	Cycle and treadmill	NE	aerobic exercise training	3 or 4 times per week	24	④⑤	4
Damon L Swift-b et al	2012	USA	Clinical Trials.gov (NCT00011193)	315	89	56.93 ± 6.4	57.0 ± 5.8	Cycle and treadmill	RH	aerobic exercise training	3 or 4 times per week	24	④⑤	7
Hajime Miura et al	2015	Japan	NR	45	47	73.1 ± 6	70.0 ± 6.9	Rubber tube and/or dumbbells	NE	resistance exercise training	2 times per week	12	①④⑤	3
Jeong-Ah Lee et al	2012	Korea	NR	8	8	54.75 ± 2.76	54.25 ± 2.91	Yoga exercise	NE	aerobic exercise training	3 times per week	16	④⑤	2
Kerrie L Moreau et al	2001	USA	NR	15	9	53 ± 7.75	55 ± 3	Walking program	RH	aerobic exercise training	Everyday	12	⑤	3
Khalid Turky et al	2013	Egypt	NR	12	13	52.9 ± 2.6	52.7 ± 2.2	Stretching and treadmill	NE	aerobic exercise training	3 times per week	8	④⑤	6
Masanori Ohta et al	2012	Japan	NR	13	13	72.2 ± 4.2	71.5 ± 7.4	Bench step	RH	aerobic exercise training	3 times per day	12	①④⑤	2
Michael Gregory et al	2012	Canada	NR	5	3	67 ± 5	62 ± 7	Isometric handgrip training	ST	aerobic exercise training	3 times per week	8	④⑤⑥	5
Mona Mohamed Taha et al	2016	Egypt	NR	23	23	48.17 ± 2.2	47.78 ± 2.59	Electronic treadmill	NE	aerobic exercise training	3 times per week	10	④⑤	3
Noushin Azadpour et al	2017	Turkey	NR	12	12	57.58 ± 4.29	56.58 ± 4.17	Treadmill	RH	aerobic exercise training	3 times per week	10	④⑤	3
Reza Nuri et al	2012	Iran	NR	14	15	58.27 ± 6.31		Walking and resistance training	NE	combined exercise training	2 times per week	15	④	5
Timothy S Church et al	2007	USA	Clinical Trials.gov (NCT00011193)	362	102	57.3 ± 6.6	57.2 ± 5.8	Cycle and treadmill	NE	aerobic exercise training	3 or 4 times per week	24	④⑤	7
Won-Mok Son et al	2017	Korea	NR	10	10	76 ± 5	74.7 ± 2	Walking and resistance training	NE	combined exercise training	3 times per week	12	①⑥	3
Won-Mok Son et al	2020	Korea	Clinical Trials.gov (NCT03919201)	10	10	67.7 ± 1.0	67.4 ± 1.1	Resistance band exercise	NE	resistance exercise training	3 times per week	12	④⑤	6

① brachial-ankle pulse wave velocity (baPWV); ② augmentation index (AIx); ③ AIx normalized to a heart rate of 75 beats per minute (AIx@75%); ④ systolic blood pressure (SBP); ⑤ diastolic blood pressure (DBP); ⑥ pulse pressure (PP)

*T* treatment, *C* control, *NR* not reported, *RH* regular lifestyle habits, *ST* sham training, *NE* no exercise

### Arterial stiffness

#### baPWV

**Fig. 2:**
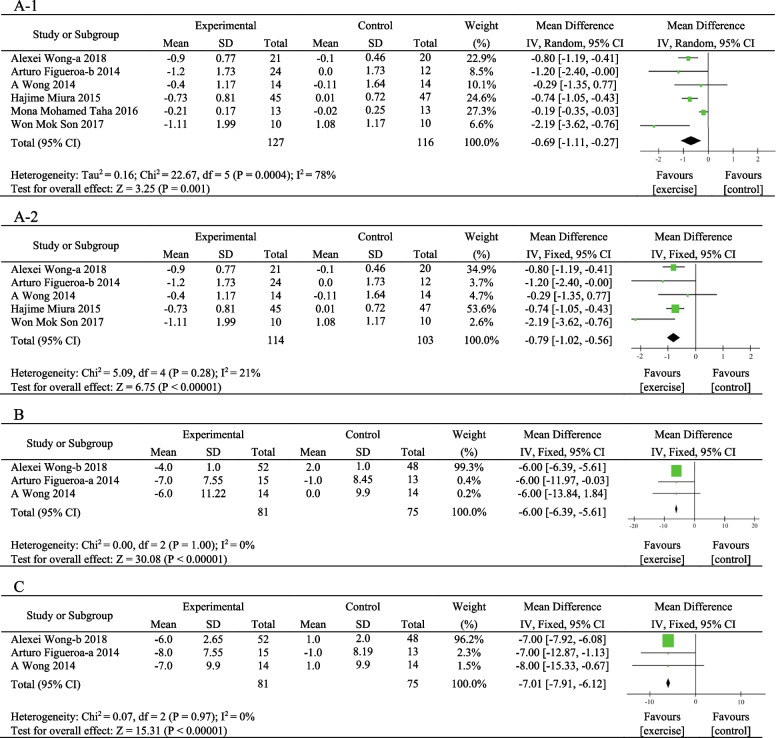
A meta-analysis of the exercise effects on arterial stiffness. **A-1** Meta-analysis of all studies, **A-2** Mona Mohamed Taha et al. removed baPWV, **B** AIx, and **C** AIx@75%

Six eligible RCTs with 243 participants [29, 30, 32, 35, 40, 41] examined the effect of exercise training on baPWV. Combining findings based on the random-effects model, we found that baPWV levels were significantly reduced in the exercise training group compared with the control group [MD = − 0.69, 95%CI (− 1.11, − 0.27), *P* = 0.001], with high heterogeneity among studies (*I*^2^ = 78%, *P* = 0.0004) (Fig. 2A-1). Sensitivity analysis was performed by excluding studies one by one, and heterogeneity decreased remarkably when the study of Mona Mohamed Taha et al. [41] was removed (Fig. 2A-2). Meanwhile, it was found that there were no significant differences in the sample size, duration of treatment, and other aspects of the six studies by tracing the original literature. The results suggested that exercise training may reduce baPWV and improve arterial stiffness in postmenopausal women with elevated blood pressure.

#### AIx and AIx@75%

Three trials involving 156 participants [32, 42, 43] reported AIx and AIx@75% as outcomes. Collectively, results from the fixed-effects model (*I*^2^ = 0%) indicated that both AIx [MD = − 6.00, 95%CI (− 6.39, − 5.61), *P* < 0.00001] (Fig. 2B) and AIx@75% [MD = − 7.01, 95%CI (− 7.91, − 6.12), *P* < 0.00001] (Fig. 2C) levels appeared substantially different in the exercise training group compared with the control group. The findings resulted in statistical significance for the pooled effects, indicating that exercise training can improve the elasticity of the arteries and arterial stiffness in postmenopausal women with elevated blood pressure.

### Blood pressure

#### SBP

**Fig. 3:**
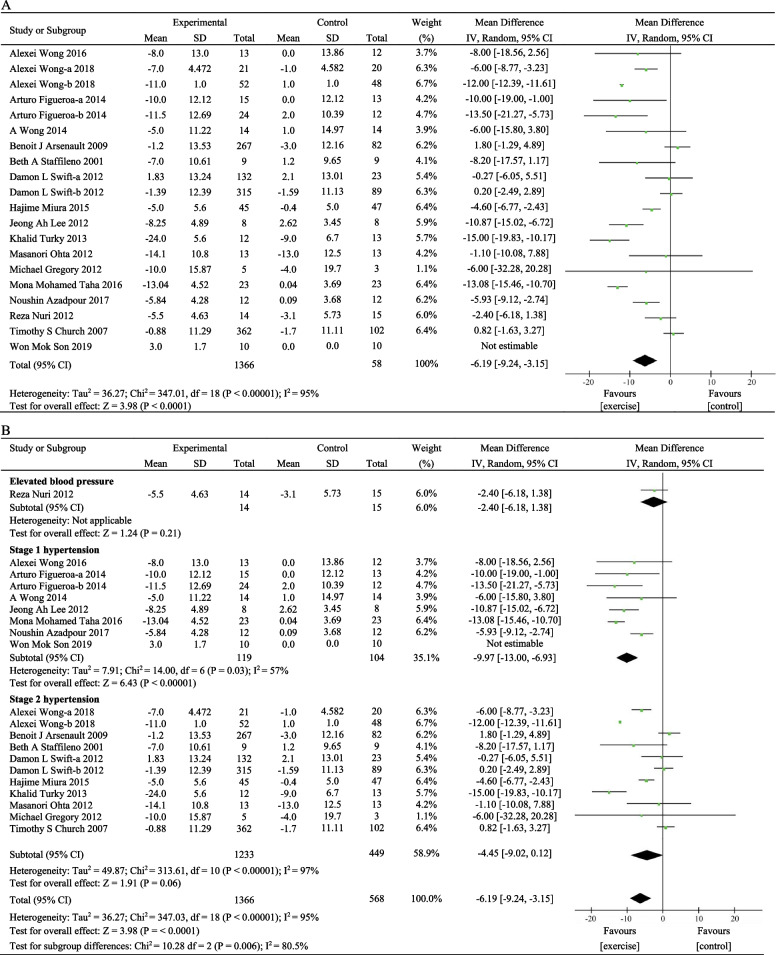
A meta-analysis of the exercise effects on SBP. **A** Meta-analysis of all studies, **B** subgroup analysis based on baseline SBP

Twenty eligible RCTs involving 1934 participants [29–34, 40–53] examined the effect of exercise training on SBP. Combined results from the random-effects model (*I*^2^ = 94%, *P* < 0.00001) indicated that the changes in SBP levels were statistically significant before and after exercise training [MD = − 6.19, 95%CI (− 9.24, − 3.15), *P* < 0.0001] (Fig. 3A). No significant effect for sensitivity was observed. According to the baseline blood pressure levels, SBP between 120 and 129 mmHg was considered elevated SBP, SBP between 130 and 139 mmHg was considered stage 1 hypertension, and SBP ≥ 140 mmHg was defined as stage 2 hypertension [36]. Subgroup analysis showed that there was moderate or high heterogeneity in each subgroup (Fig. 3B). Combined results from the random-effects model (*I*^2^ = 57%, *P* = 0.03) showed that SBP levels changed after exercise training [MD = − 9.97, 95%CI (− 13.00, − 6.93), *P* < 0.0001] in the population with stage 1 hypertension, indicating a positive effect of exercise training on reducing blood pressure in postmenopausal women with stage 1 hypertension.

#### DBP

**Fig. 4:**
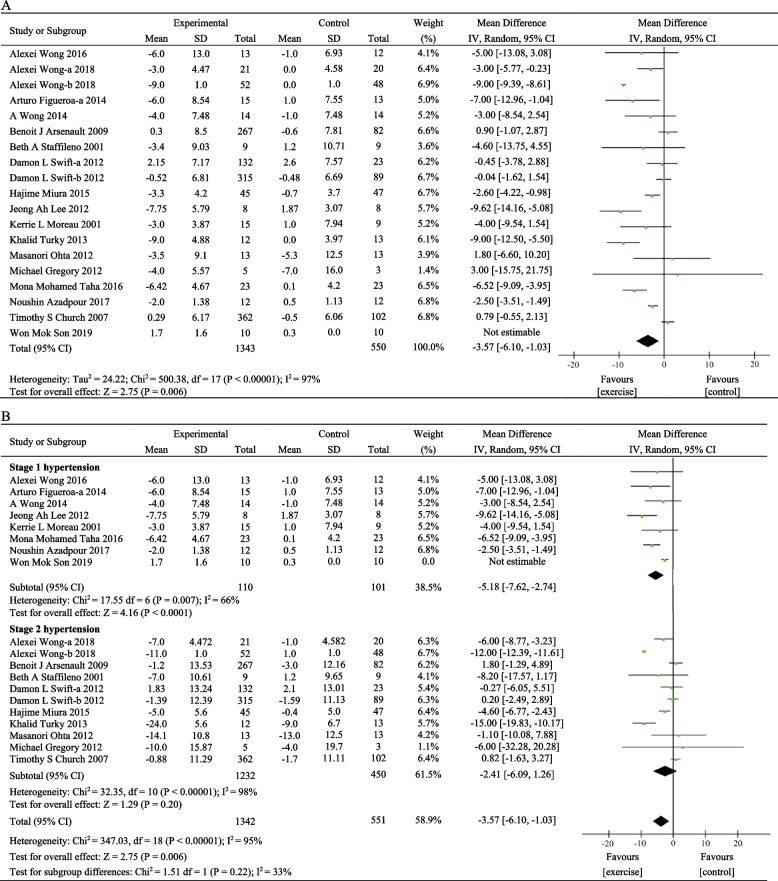
A meta-analysis of the exercise effects on DBP. **A** Meta-analysis of all studies, **B** subgroup analysis based on baseline DBP

Nineteen eligible trials reported DBP as an outcome [29–34, 41–50, 52–54], and the combined results from the random-effects model showed that exercise training had a positive effect on the reduction of DBP [MD = − 3.57, 95%CI (− 6.10, − 1.03), *P* = 0.006). These studies included a total of 1893 participants, with high heterogeneity between studies (*I*^2^ = 96%, *P* < 0.00001) (Fig. 4A). No significant effect for sensitivity was observed. Meanwhile, we used baseline DBP as the criteria for subgroup analysis. DBP < 80 mmHg was considered as elevated blood pressure, DBP between 80 and 89 mmHg was considered stage 1 hypertension, and DBP ≥ 90 mmHg was defined as stage 2 hypertension, and individuals with SBP and DBP in 2 categories should be designated to the higher blood pressure category [36]. Subgroup analysis revealed that heterogeneity in each subgroup remained high (Fig. 4B). The results suggested that exercise training may reduce DBP, but the reduction value is smaller than that of SBP.

#### PP

**Fig. 5:**
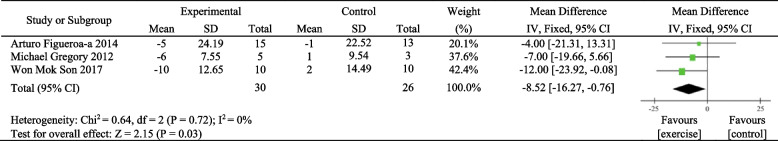
A meta-analysis of the exercise effects on PP

Three eligible trials [35, 43, 49] examined the effect of exercise training on PP. Collectively, results from the fixed-effects model indicated that PP levels were reduced in the exercise training group compared with the control group [MD = − 8.52, 95%CI (− 16.27, − 0.76), *P* = 0.03), with low heterogeneity between studies (*I*^2^ = 0%, *P* = 0.72) (Fig. 5). The results suggested that exercise may reduce PP, indicating that exercise training can potentially improve the elastic function of arteries.

### Adverse events

In all, 22 trials including 1978 patients provided no detailed data on adverse events except Khalid Turky et al. [47]. In this study, one participant had acute back pain at the end of training, which focused on the efficacy and safety of stretching and treadmill walking.

### Quality assessment

**Fig. 6:**
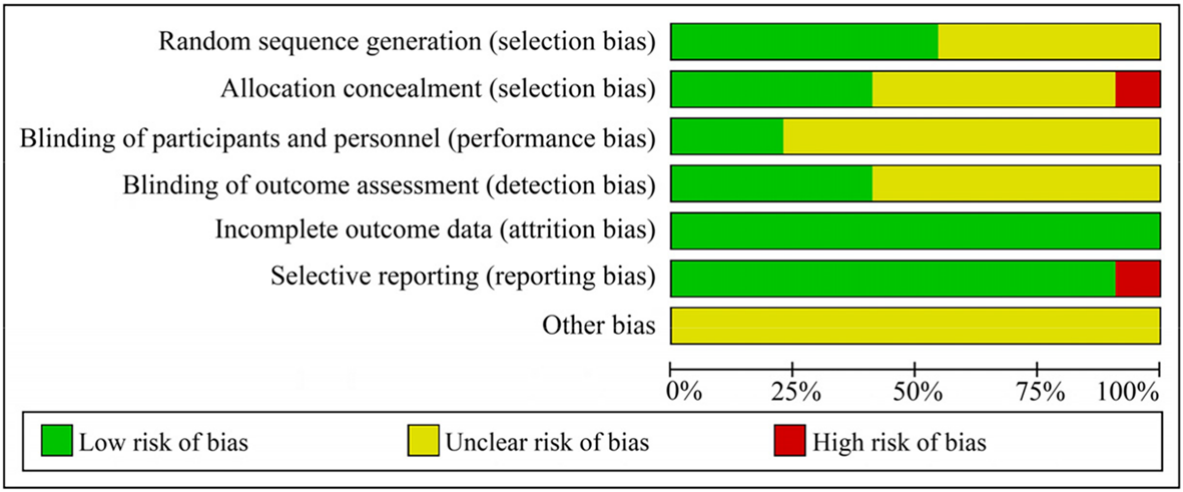
Risk of bias of included studies

The qualities of included RCTs were evaluated by the Jadad scale, and the results were summarized in Fig. 6. A total of 12 eligible trials were found to be of high quality. The quality of results ranged from 2 to 7, with an average score of 4.2. All of the studies were randomized, and half of them reported the method used for randomization [29, 33, 40–42, 44, 45, 47, 49, 52, 53]. For blinding, seven articles blinded the observer during outcome assessment, but specific blinding methods were not available [40, 42, 44, 45, 47, 52, 53]. Furthermore, dropouts were listed and described in the one of articles [47].

### Publication bias assessment

**Fig. 7:**
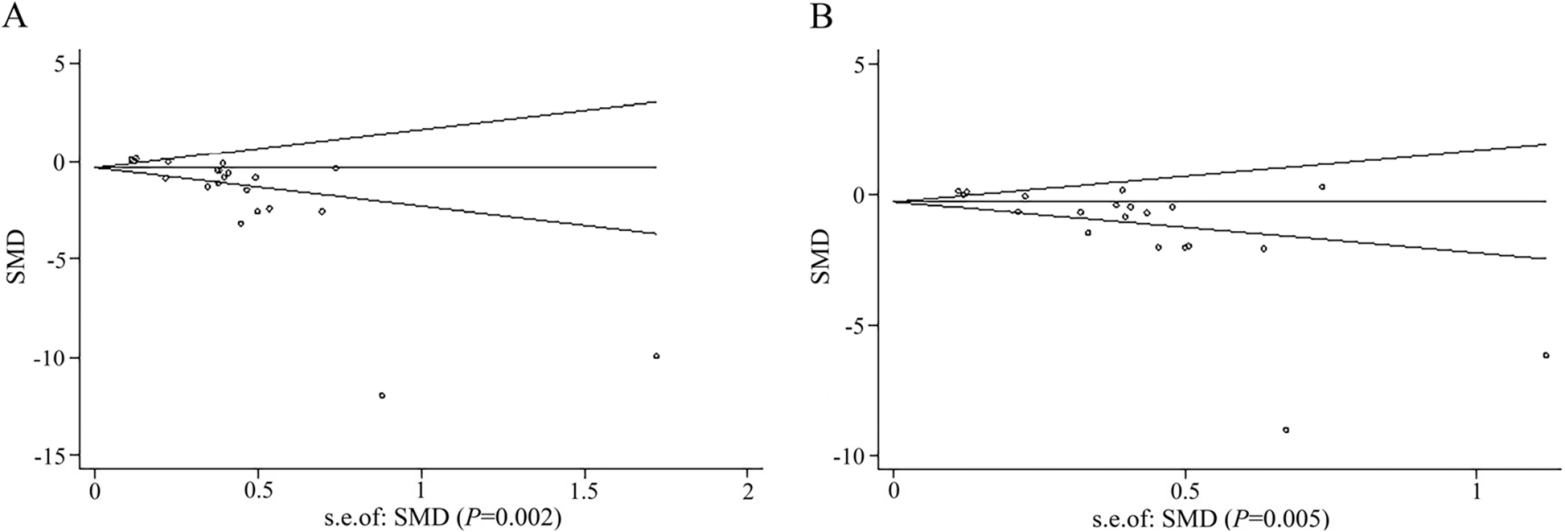
Publication Bias assessment of included studies. Begg’s funnel plot pseudo 95% confidence limits of **A** SBP and **B** DBP

Funnel plots were conducted for SBP and DBP to analyze the potential publication bias of the included studies via Begg’s test (Fig. 7). The results revealed that most of the study sites in the funnel plot were located within 95%CI range, but the shape formed by the study sites was not completely asymmetric, suggesting a potential publication bias of these studies in SBP (*P* = 0.002) and DBP (*P* = 0.005), which may be related to the small sample size, different treatment courses, and low quality of included studies.

## Discussion

This systematic review and meta-analysis presented a comprehensive overview of several exercise training on arterial stiffness and blood pressure in postmenopausal women with elevated blood pressure reported in 22 RCTs. Different types of aerobic exercise and/or resistance exercise training, such as walking, running, climbing stairs, swimming, resistance band exercises, whole-body vibration, rubber tube, dumbbells, isometric handgrip training, stretching exercises, bench step exercise, yoga, high-intensity interval training, and combined exercise training were included in our study. There was no restriction on the frequency and duration of training. Overall, current evidence may allow recommendations about exercise training for arterial stiffness and blood pressure in postmenopausal women with elevated blood pressure. However, the study findings should be interpreted cautiously given the substantial heterogeneity between available publications and the low quality of the majority of the included studies.

The baPWV is determined by the conduction time between the brachial artery and the ankle regional distance between these two segments. BaPWV reflects the structural and functional stiffness of the arterial wall and is often used to evaluate the level of arterial stiffness in clinical practice, with the characteristics of simple operation, repeatability, and non-invasiveness [55]. Studies showed that both aerobic and resistance exercise training may increase the diameter of the major arteries and help reduce peripheral arterial stiffness, especially the brachial or femoral arteries [56]. The beneficial effect of exercise training on the arteries may be related to the effect of increased blood flow on the endothelium, resulting in structural remodeling and the reduction of vascular smooth muscle tone [57]. The baPWV is primarily determined by central arterial stiffness and is generally higher than the PWV of the upper and lower limb arteries indicating that baPWV may be affected by the peripheral arterial stiffness [58–60]. However, the baPWV is an appropriate option for clinical applications. BaPWV reflects vascular stiffness, while AIx is a measure of wave reflection. The AIx is calculated from parameters measured by pulse waves and associated with arterial stiffness [61, 62], and AIx measurements are standardized to a heart rate of 75 bpm [63]. Higher AIx were associated with a higher risk of hypertension [64]. The presence of hypertension is one of the main determinants of the accelerated progression of aortic stiffness in treated hypertensive patients [65]. Arterial stiffness is related to the early return of reflex waves and the increase of amplitude, which is one of the main factors of abnormal blood pressure [66]. Previous studies showed that higher baPWV is closely related to the elderly [67, 68], higher mean arterial blood pressure [69], and hypertension [70]. Besides, elderly women often have a high wave reflection [71]. Arterial stiffness is an indicator of early adverse structural and functional changes in the arterial wall and has been proven as an independent predictor for cardiovascular morbidity and mortality [72–74]. Studies found that DBP levels were strongly correlated to AIx [75, 76]. Peripheral pulse pressure also provided a surrogate measure of arterial stiffness, which was considered as an independent predictor of cardiovascular outcomes in hypertensive patients [77].

The results of our systematic review and meta-analysis suggested that the effect of exercise training was superior to no exercise or sedentary in improving arterial stiffness and blood pressure in postmenopausal women with elevated blood pressure. Previous studies have investigated the exercise effects on arterial stiffness and blood pressure in different subjects such as healthy people [77], adults [78], overweight or obese populations [79], and chronic kidney disease [80]. We found that the baPWV of the intervention group was reduced by 0.79 m/s more than that of the control group. At present, there is no unified standard for the definition of minimum clinically important difference (MCID) of arterial stiffness. The findings should be considered meaningful compared with other results [35, 81, 82]. Moreover, heterogeneity decreased remarkably when the study of Mona Mohamed Taha et al. [41] was removed. The average age of participants in Mona Mohamed Taha et al. [41] was less than 50 years old, while patients were all over 50 years old in the remaining studies, suggesting that age might be the source of heterogeneity. Data from the Kailuan study cohort involving 940 participants [83] showed that aerobic exercise had an acute positive effect on arterial stiffness and provided evidence of a greater reduction in arterial stiffness in individuals without hypertension than in those with hypertension. While a meta-analysis [27] suggested that PWV decreased after aerobic exercise training. A review [26] of 10 RCTs indicated that resistance training stand-alone did not elicit changes in the prognosis of cardiovascular diseases in healthy subjects. Similarly, the same result was observed in another study [27]. In our systematic review and meta-analysis, the effect of resistance exercise training [30] and combined exercise training [35] on arterial stiffness was assessed in only one study, respectively, leading to a reduction in the reliability and generalizability of our findings. A meta-analysis involving 21 RCTS found that a combination of aerobic and resistance training interventions may reduce the beneficial effect on arterial stiffness, but did not appear to differ significantly with aerobic training alone [82]. We found that exercise training was beneficial for lowering blood pressure. Sensitivity analysis and subgroup analysis of SBP and DBP failed to reduce heterogeneity. Blood pressure was greatly affected by multiple factors such as small sample size, environment, measurement tools, measurement methods, different treatment courses, and low quality of the included studies, which may be the sources of high heterogeneity. Similar to our results, a systematic review found that exercise training was associated with a reduction in SBP and DBP in menopausal and postmenopausal women with elevated blood pressure [84]. Our findings indicated that exercise training could decrease SBP in postmenopausal women with elevated blood pressure, especially in those with stage 1 hypertension.

Oxidative stress and inflammation are the main mechanisms that cause arterial stiffness [85, 86]. The mechanisms associated with hypertension in postmenopausal women are initiated by the loss of endogenous estradiol and changes in other reproductive hormones. Endothelial dysfunction is an important precursor of cardiovascular disease. Importantly, the decline in endothelial function is independent of age but may be associated with the deficiencies of estrogen and L-arginine in postmenopausal women [87]. Postmenopausal women suffer from a series of changes that include the deficiency of estrogen, increment in proinflammatory cytokines production, strengthening of the oxidative stress response, deduction on L-arginine production, reduction of NO bioavailability, and inferior arterial response to acetylcholine, which are considered to be major causes of vasodilation impairment and endothelial dysfunction [88–90]. Potential therapeutic targets include enhancing L-arginine bioavailability and estrogen receptor activation to prevent endothelial dysfunction in postmenopausal women [91]. Aerobic exercise training is beneficial to lowering blood pressure, with a focus on improvements in cardiovascular autonomic control [92] and baroreflex sensitivity [93]. High-intensity resistance training may strongly stimulate the activity of the sympathetic nervous system, leading to increased blood pressure and aggravation of arterial stiffness [94]. Therefore, the long-term benefits of resistance training for postmenopausal women are still worth exploring.

The underlying mechanism of exercise training on arterial stiffness and blood pressure involves multiple pathways. Exercise training plays an active role in increasing the shear stress in the artery wall, enhancing endothelial cell integrity through remodeling, and improving NO bioavailability [95]. Moreover, exercise training is also associated with increased endothelin-1 and NO, enhanced endothelial function, reduced peripheral vascular resistance, and improved arterial stiffness [35]. Other potential mechanisms include improved autonomic nerve function and baroreflex sensitivity, reduced oxidative stress, and lipid deposition [96].

To appropriately interpret our results, several limitations need to be understood. Firstly, studies on aerobic training accounted for the majority of the obtained RCTs, possibly masking the effect of resistance training and combined training on the results. Secondly, the interventions in our study determined that exercise training was difficult to perform using blinding, thus affecting the methodological quality of this review and potential publication bias. However, due to the quality assessment and publication bias evaluation, the impact of the aforementioned potential conflicts on the procedures or results of this systematic review and meta-analysis may be reduced. Thirdly, heterogeneity in some outcomes remained undiminished even after differences in the patient’s characteristics, exercise type, and duration had been considered. The applicability of this systematic review and meta-analysis to the broader patient population may be limited given that most studies involved were conducted in specific countries. Therefore, further studies are required to confirm our results and determine the mechanisms behind the connection between exercise training and arterial stiffness and blood pressure in postmenopausal women with elevated blood pressure.

## Conclusions

To conclude, this systematic review and meta-analysis determined a positive association between exercise training and arterial stiffness and blood pressure in postmenopausal women with elevated blood pressure. Existing reviews did not provide more granular evidence in terms of different exercise patterns (e.g., type, quantity, and intensity), and therefore this should be a priority for future studies. Methodologically robust RCTs are required to determine causal links between exercise training and arterial stiffness and blood pressure, and whether this should be by aerobic training or resistance training or a mixture of the two.

### Supplementary Information

Additional file 1. PRISMA Checklist.

Additional file 2. Search strategy.

## Data Availability

All data generated or analyzed during this study are included in this published article. Other data supporting the results of this study are available from the corresponding author upon reasonable request.
