# Supplementary material for: Effectiveness of exercise training on arterial stiffness and blood pressure among postmenopausal women: a systematic review and meta-analysis
Source: Syst Rev. 2024 Jul 2;13:169. doi: 10.1186/s13643-024-02589-y (PMC11221034; doi:10.1186/s13643-024-02589-y)
Supplement: Supplementary file 2 — Additional file 2. Search strategy. [file 13643_2024_2589_MOESM2_ESM.pdf]

## Search strategy

#1 postmenopausal [MeSH Terms]
#2 postmenopausal [Title/Abstract]
#3 menopause [MeSH Terms]
#4 menopause [Title/Abstract]
#5 #1 OR #2 OR #3 OR #4
#6 stiffness [MeSH Terms]
#7 stiffness [Title/Abstract]
#8 blood pressure [Title/Abstract]
#9 blood pressure [Title/Abstract]
#10 #6 OR #7 OR #8 OR #9
#11 exercise [MeSH Terms]
#12 exercise [Title/Abstract]
#13 training [MeSH Terms]
#14 training [Title/Abstract]
#15 #11 OR #12 OR #13 OR #14
#16 #5 AND #10 AND #15
